# An exploration of treatment seeking behavior of women experienced infertility and need for services in rural India

**DOI:** 10.3389/frph.2022.978085

**Published:** 2022-08-30

**Authors:** Shraboni Patra, Sayeed Unisa

**Affiliations:** ^1^Department of Population and Development, International Institute for Population Sciences (IIPS), Mumbai, India; ^2^Department of Biostatistics and Epidemiology, International Institute for Population Sciences (IIPS), Mumbai, India

**Keywords:** infertility, treatment, behavior, health services, primary data, rural, India

## Abstract

**Background:**

To make informed decisions on fertility treatment, couples need to understand the treatment options available to them. A wide range of treatment options is available from the traditional and biomedical service providers in India. There is a dearth of research to find out factors that influence the treatment-seeking behavior of couples, particularly in rural areas.

**Objectives:**

The study aimed to document the treatment-seeking behavior of women for their infertility problems. Further, the research focused on the socio-economic determinants affecting allopathic treatment-seeking of women and the services needed for couples experiencing infertility in rural India.

**Methods:**

The study is cross-sectional. Primary data were collected from the two high infertility prevalence districts. Complete mapping and listing were carried out to identify the eligible respondents. A total of 159 ever-married women (20–49 years) out of 172 identified women were interviewed. Bivariate and multivariate analyses were performed.

**Results:**

Among 159 interviewed women, only three did not seek any kind of treatment. Of the 156 women, 63, 65, and 28 women (mutually exclusive) received first, second and third-order treatment, respectively. The number of women decreased in the succeeding phases of infertility. Women aged above 35 years, were significantly less (OR = 0.310, *p* < 0.05) compared to women aged below 30 years to receive allopathic treatment. The use of allopathic treatment was significantly three times higher among women who were educated (OR = 3.712, *p* < 0.01) and two times higher among those who were exposed (OR = 2.217, *p* < 0.5) to media. Further, for those who had felt the treatment was necessary, about 30, 44, 10, and 19% mentioned that due to unaffordability, inaccessibility, or inconveniences they couldn't consult allopathic treatment.

**Conclusions:**

Timely diagnosis and appropriate treatment play important role in infertility management. Women who are more educated and are exposed to media tend to consult allopathic treatment. Similarly, time and money spent on care vary significantly and independently by type of treatment and socioeconomic factors. There is a need for mandatory insurance coverage for infertility treatment enacted by the state government. In addition to the public services, the private sector and the traditional healers are both important alternative sources of first help.

## Background

Delayed childbearing has become increasingly socially acceptable, and a considerable amount of favorable media attention has been given to older mothers. Current advances in assisted reproductive techniques (ART) may have caused misconceptions about the possibility of manipulating female fertility. Yet none of these advances can fully compensate for the age-related decline in female fertility (1, 2). According to World Health Organization, Infertility is a disease of the male or female reproductive system defined by the failure to achieve a pregnancy after 12 months or more of regular unprotected sexual intercourse (3). Infertility can be considered in terms of *primary* infertility, where the woman has never conceived and *secondary* infertility where she has conceived once but not subsequently despite efforts to become pregnant. Primary infertility is defined as childlessness while secondary infertility is considered as the inability to have an additional live birth for a porous woman (4). Infertility affects millions of people of reproductive age worldwide and has an impact on their families and communities. Estimates suggest that between 48 million couples and 186 million individuals live with infertility globally. Due to the high cultural premium placed on childbearing in many countries, infertility often poses serious social problems for couples (5).

Fertility treatments available to couples are often complex (6). To make informed decisions on fertility treatment, couples need to understand the treatment options available to them. The large volume of research on fertility treatments, which is often of poor quality, makes it difficult to access reliable, relevant, and readable information. This makes the emotional decision-making process even more of a challenge (7). Further, depending on the type of treatment, diagnosis, and medication available to treat fertility complications among women (8), expenses also vary to a large extent. To treat infertility, a wide range of treatment options are available from the traditional and biomedical service providers in India (9). Some factors influence the treatment-seeking behavior of women, such as the willingness of infertile couples to seek treatment, social and emotional repercussions during ongoing treatment, accessibility of infertility care (10) and affordability of the expenses of infertility treatment (11–14).

Infertility is not high on the agenda of policymakers (15), as it is not life-threatening and in a densely populated country like India, the problem of infertility and childlessness is not much annoying when other health-related issues are taken care of. Again, there are so many factors that influence the treatment-seeking behavior of women and the type of treatment sought by them (16). Literature shows that the importance of modern allopathic treatment in treating infertility is well accepted and the success rate of allopathic treatment is found significantly higher as compared to the other types of treatment (17, 18). Besides, AYUSH (i.e. Ayurveda, Yoga and Naturopathy, Unani, Siddha and Homeopathy are the six systems of medicine, prevalent and practiced in India) has been receiving much importance in infertility treatment due to its low cost and easy availability in remote areas (19–21).

Hence the present research aims to document the coping strategies, particularly the treatment-seeking behavior of women for their infertility problems in rural West Bengal. Further, the present research has tried to focus on the factors, particularly the socio-economic determinants affecting allopathic treatment-seeking of women. The study also throws some light on the services needed for couples experiencing infertility in the study areas. This paper seeks to address this lacuna by specifically focusing on infertility in rural India.

**Figure 1:**
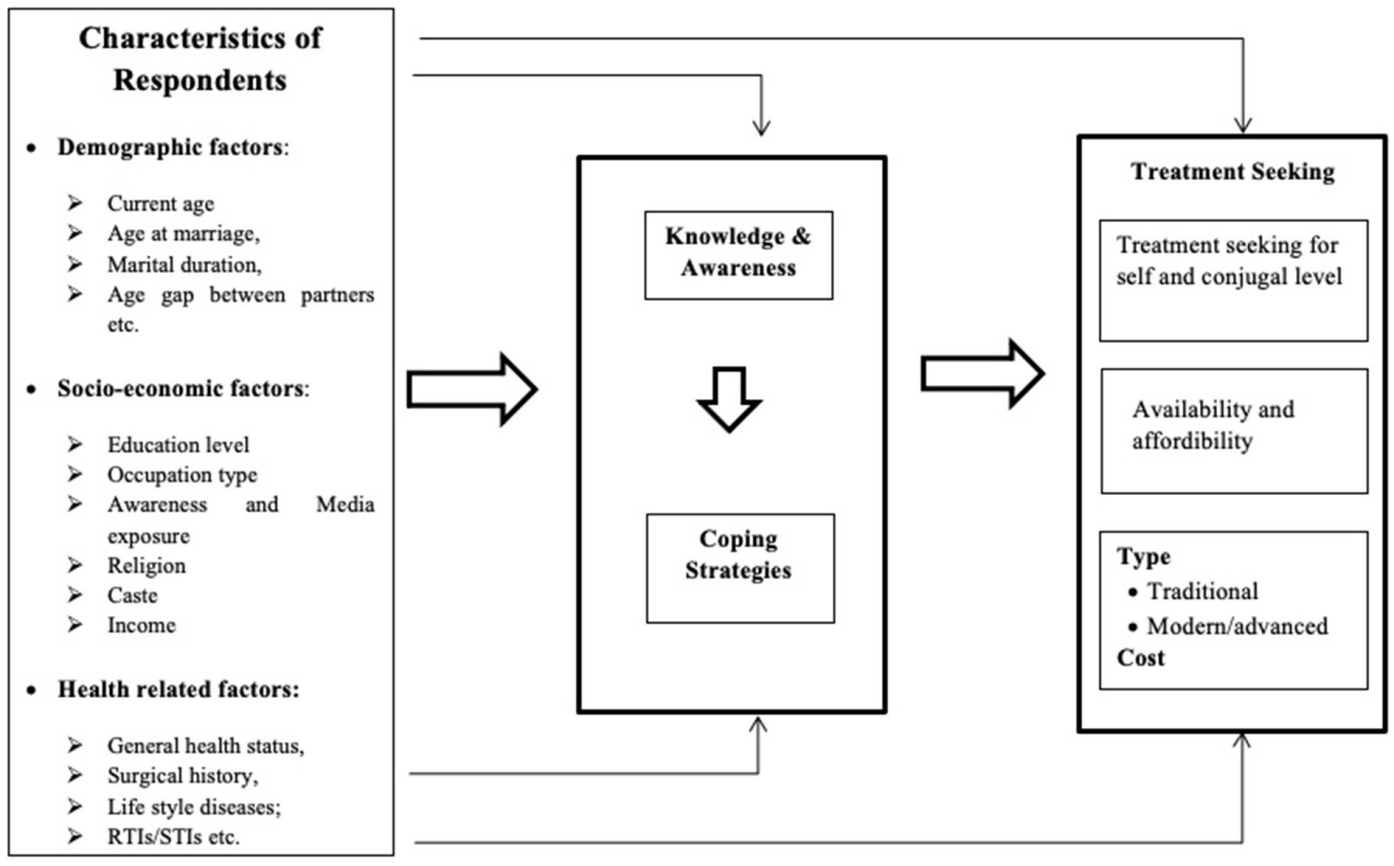
Conceptual framework.

A conceptual framework was developed to gain more insights into the study (Figure 1).

## Methods

### Study design, area, and period

The present study is observational and cross-sectional for which primary data were collected in 2015–16. Two high infertility prevalence districts i.e., Purab Medinipur (17%) and Dakshin Dinajpur (19.4%) of West Bengal (14.1%), an Indian state with a low fertility rate (22), were selected as the study area. To get the desirable sample, one block from each identified district was selected. Care was also taken in selecting mainly rural blocks. These two blocks contain a heterogeneous population. From these selected blocks, one village with a primary health center (PHC), one village with at least one sub-center (SC), and one village without any government health facility were selected purposively. Therefore, from the two selected blocks, a total of six villages (three villages from each block) were selected.

### Study sample and inclusion criteria

Complete mapping and listing were carried out to identify the eligible respondents. Data were collected through semi-structured and structured questionnaires. A total of 159 ever-married women (20–49 years) out of 172 identified women who ever had experienced infertility were interviewed. Hence, the estimated response rate was 92.4%. The inclusion criteria of the respondents were: ever experienced infertility problems, both the husband and wife necessarily not using any contraception, not sterilized (women were not lactating and not pregnant) and received treatment/ advice for their problem of infertility. The informed consent of the participants was obtained before collecting the data.

In the selected villages, mapping, and complete house listing were carried out. All the households were screened primarily for the following groups of women:

***Childless women*** were selected based on the following criteria: women aged 20–49 years; were currently married, were residing with their husbands and marital duration of at least 2 years; women who had never given a live birth; were not using contraception; and were not sterilized (were not lactating and not pregnant).

***Women who ever conceived and ever experienced problems in getting pregnant*** were also selected based on the following criteria: women aged 20–49 years; women who were currently married and were residing with their husbands; and women who had ever experienced infertility problem and sought treatment.

Among the identified women, those who were voluntarily childless for a few years after marriage didn't seek any treatment for infertility (both husbands and wife) and were normally conceived were excluded from the study. Those women (or husband) who had ever experienced infertility problems, received treatments, and had a live birth, and those women who had at least one successful conception and were experiencing infertility problem (secondary infertility) were also considered for an interview.

### Study variables

The background variables, used to represent the socio-economic characteristics of the respondents and their categories were: respondents' current age (<26, 26–30, 31–35, 36–40, and >40 years); age at marriage (<18 and = >18 years); marital duration (<6, 6–10, 11–15, 16–20, >20 years); age gap between husband and wife (1–3, 4–6, 7–9, >9 years); level of education (illiterate, 1–4 years/primary, 5–8 years/middle or upper primary and 9–10 years/secondary and higher secondary, >12 years/graduate and above); husband's level of education (as for the respondents); respondent's media exposure (not exposed and exposed, i.e., whether she had received any information on fertility and treatment-related issues from any kind of media); respondent's work status (never worked and ever worked); religion (Hindu and Muslim); caste (Scheduled caste/SC, Scheduled tribe/ST and Other Backward Caste/ OBC and General/open); monthly income (<=5,000, 6,000–10,000, 11,000–15,000, >15,000 rupees); type of treatment women received (allopathic, AYUSH, traditional/home remedies/religious remedies; type of village women belong to (village with PHC, village with SC, village with no government health facility).

### Statistical analyses

Bivariate and multivariate analyses were performed. Principal Component Analysis (PCA) was carried out to create the ‘Wealth Index' (WI). This was a composite index based on information on household ownership of assets, amenities, fuel use and other wealth. The first principal component generated by PCA was used to assign the weights for these assets. The reliability of the index was tested by Cronbach‘s alpha values (0.896), which suggested that the computed index was reliable. The value of the composite index was divided into three equal parts (low, medium, and high) for subsequent analysis. Hence, the variable “Wealth Index” had three categories, i.e. poor, middle, and rich.

The logistic regression model was used to analyze the effect of selected socio-economic factors on allopathic treatment received by the respondents. Binary logistic regression was used to estimate the adjusted effect (odds ratio/OR) of background characteristics on treatment received. Statistical Packages for the Social Sciences (SPSS), version 20 (IBM Corp, Armonk, New York), and Stata V.13.0 (Stata Corp, College Station, Texas, USA) were used for data analysis.

## Results

### Profile of the respondents

Table 1 represents the socioeconomic profile of the respondents. One-third of the respondents were aged between 26 and 30 years. The mean age of the respondents was 32 years. About 29 percent of the respondents were married below the legal age (i.e., 18 years). The mean age at marriage was 19.5 years. A considerable percentage (28%) of women had a marital duration of more than 15 years. Hence, the mean duration (years) of marriage was 12 years. A huge proportion (43%) of the respondents had more than 6 years of age gap with their husbands.

**Table 1:** Percentage distribution of respondents by socio-economic characteristics.

**Characteristics**	**Percent**	**Women (*n*)**
**Age of women in years**		
<26	17.6	28
26–30	33.3	53
31–35	20.8	33
36–40	17.6	28
>40	10.7	17
Mean age in years	31.6	159
**Age at marriage in years**		
<18	28.9	46
≥18	71.1	113
Mean age at marriage in years	19.5	159
**Marital duration in years**		
<6	18.2	29
6–10	35.8	57
11–15	17.6	28
16–20	14.5	23
>20	13.8	22
Mean duration of marriage in years	12.1	159
**Age gap between partners in years**		
1–3	9.4	15
4–6	47.8	76
7–9	36.5	58
>9	6.3	10
**Education level of women**		
Illiterate/ uneducated	23.3	37
Primary	25.8	41
Middle/upper primary	28.3	45
Secondary and higher secondary	18.2	29
Graduate/Post-graduate/Diploma	4.4	7
**Exposure to media**		
Not at all	29.6	47
Yes, almost every day	34.6	55
Yes, At least once a week	21.4	34
Less than once a week	14.5	23
**Occupation**		
Never worked	59.1	94
Agricultural	3.8	6
Service	3.8	6
Bidi-making	13.8	22
Jari/Stitch	8.2	13
Others	11.3	18
**Religion**		
Hindu	86.8	138
Muslim	13.2	21
**Caste**		
Scheduled caste	14.5	26
Scheduled tribe	3.8	5
OBC	15.1	23
General/other	66.7	105
**Monthly income in rupees**		
≤ 5,000	24.5	39
5,001–10,000	33.3	53
10,001–15,000	15.1	24
15,001–20,000	14.5	23
≥20,001	12.6	20
**Total**	**100.0**	**159**

### Treatment received in different orders

In other words, to receive a second or subsequent treatment, every respondent must have received the first treatment (Table 2).

**Table 2:** Percentage of women received treatment in different orders and conceived.

**Treatment received**	Women (*n*)*	**Percent**	**Able to conceive**	
			**Women**	**Percent**
No treatment	3	1.9	-	-
One treatment only	63	39.6	36	57.1
Two treatments only	65	40.9	28	43.1
More than two treatments	28	17.6	3	10.7
Total	159	100.0	67	42.1

*Mutually exclusive.

Among 159 interviewed women, only three did not seek any kind of treatment. Of the 156 women who sought treatment, 63 women had sought treatment only one time. Of these 63 women, 36 women (57.1%) were conceived, and 27 women discontinued the treatment. Of the remaining 93 women (i.e., 156–63), 65 sought treatments twice (two times only) and 28 women sought treatment more than two times. After two treatments, from 65 women 28 conceived (i.e., conception rate 43.1%), and 37 women discontinued further treatment. The remaining 28 women received three or more treatments, and only three were conceived after multiple treatments.

### Received infertility treatment in different phases and different orders

**Figure 2:**
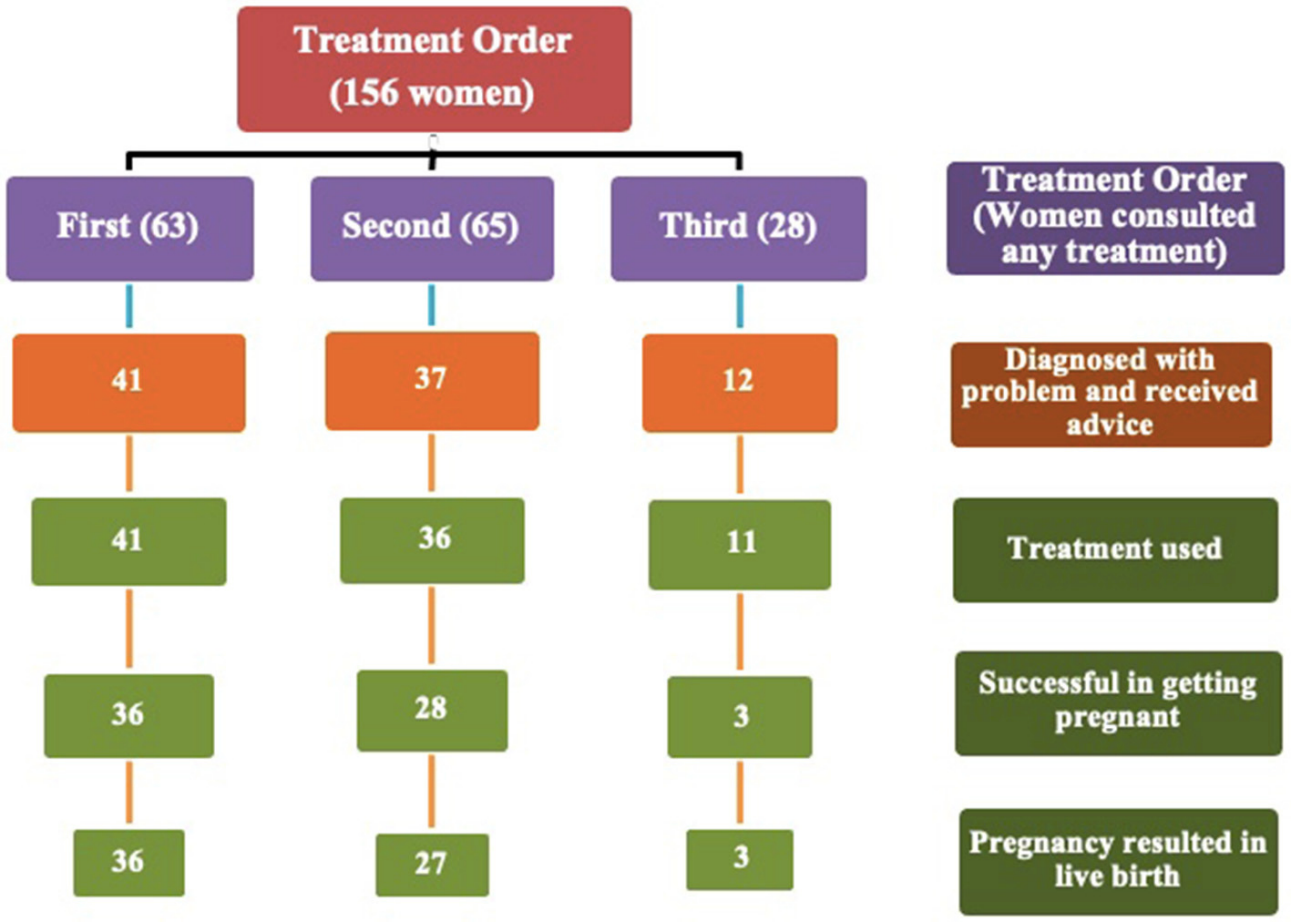
Number of women received treatment in different phases and orders.

Figure 2 represents the total number of respondents in different phases of treatment and different treatment orders. It is found that there were 63, 65, and 28 women (mutually exclusive) who received first, second and third-order treatment respectively. The present research also shows that the number of women decreased in the succeeding phases of infertility treatment (like consulting a treatment, being diagnosed with the problem, receiving advice, using the treatment, successful in conceiving and successful outcome of pregnancy or live birth). Hence, the flowchart depicts that only a few respondents who started with consulting a treatment ended up with a successful treatment outcome or had a live birth (36, 27 and 3 in first, second and third-order treatment respectively). The number of women who delivered a live birth was almost equal to the number of women who got pregnant at the end of first and third-order treatment (except for second-order treatment).

### Types of treatment received in orders

Table 3 shows the percentage of women (mutually exclusive) who received one, two and more than two treatments according to their types. The percentage of women who received traditional, home remedies and religious treatment were higher (37%) among those who received two treatments, whereas a higher percentage of women (39%) received AYUSH treatment among those who received more than two treatments.

**Table 3:** Percentage of women received different types of treatments in orders.

**Treatment type**	**Only one treatment**	**Percent**	**Two treatments**	**Percent**	**More than two treatments**	**Percent**	**Women (*n*)**
Allopathic	27	42.9	30	46.2	9	32.1	66
AYUSH	20	31.7	11	16.9	11	39.3	42
Traditional/ home remedies/religious	16	25.4	24	36.9	8	28.6	48
Total	63	100	65	100	28	100	156

### Received allopathic treatment according to the socioeconomic characteristics

Table 4 represents the percentage of women who received modern allopathic treatment according to their socio-economic characteristics. Women of the older age group (above 35 years) were significantly less (OR = 0.310, *p* < 0.05) compared to women of younger age (below 30 years) to receive allopathic treatment. The use of allopathic treatment was also significantly found three times higher among women who were educated (OR = 3.712, *p* < 0.01) and two times higher among those who were exposed (OR = 2.217, *p* < 0.5) to media as compared to their counterparts. The percentage of women who received allopathic treatment was also found higher among women from general caste (62%), Hindu religion (57%) and middle (55%) and rich (83%) economic status.

**Table 4:** Percentage of women received modern allopathic treatment for infertility problem by their background characteristics.

**Background characteristics**	**Women (%)**	**Odds ratio** **(Exp β)**	**95% CIs for Exp (**β**)**	
			**Lower**	**Upper**
**Age (years)**				
Below 30^®^	65.7	1		
30 to 35	61.4	0.883	0.343	2.274
Above 35	35.6	0.310*	0.113	0.854
**Education**				
Illiterate/uneducated^®^	18.9	1		
Educated	67.2	3.712**	1.260	10.929
**Media exposure**				
Not exposed^®^	25.5	1		
Exposed	68.8	2.217*	0.526	9.350
**Work status**				
Never worked^®^	62.8	1		
Ever worked	46.2	0.615	0.282	1.341
**Caste**				
SC/ST/OBC^®^	44.4	1		
General/Open	61.9	2.398	0.932	6.171
**Religion**				
Hindu^®^	57.2	1		
Muslim	47.6	1.592	0.456	5.559
**Wealth index**				
Poor^®^	30.2	1		
Middle	54.7	1.284*	0.315	5.243
Rich	83.0	3.427	0.742	15.829
**Total**	**56.0**			

Significance level: ** p < 0.01, * p < 0.05, ^®^ Reference category.

### Reasons behind not consulting allopathic treatment

Women who didn't consult allopathic treatment were asked to mention the reasons why did they do so. About 70% of women mentioned they did not know where to go, about 49% said they wanted to wait for natural pregnancy and about 54% responded that they didn't feel that treatment was necessary. Further, among those who felt the treatment was necessary, about 30, 44, 10, and 19% mentioned that due to unaffordability, inaccessibility, or inconveniences like no time or no one at home etc. they didn't consult allopathic treatment (Table 5).

**Table 5:** Reasons behind not consulting allopathic treatment among those who did not consult allopathic treatment.

Reasons for not consulting allopathic treatment*	**Percent**	**Women (*n* = 70)**
Expensive could not afford	30.0	21
Too far/ no transportation	44.3	31
Timing/other inconveniences	10.0	7
Family members did not allow	18.6	13
Did not know where to go	70.0	49
Waiting for a natural pregnancy	48.6	34
Did not feel treatment necessary	54.3	38
Others	4.3	3

*Multiple answers received.

### Services needed for couples experiencing infertility in the study areas

**Figure 3:**
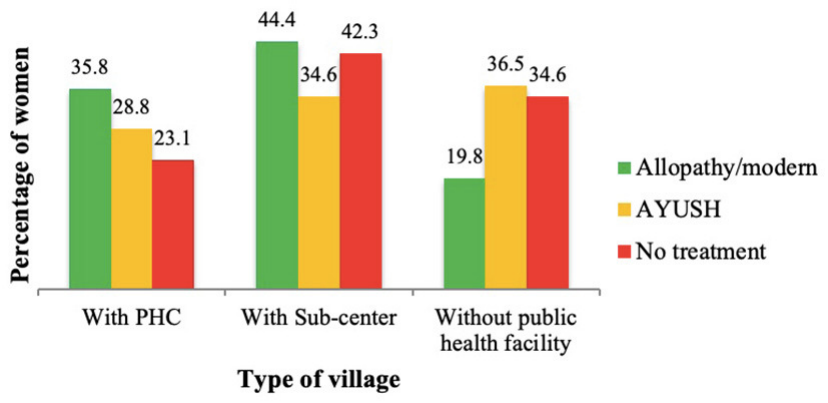
Percentage of women who received treatment according to the type of village.

The present research has shown that advanced/modern allopathy treatment received by the respondents was higher in the villages with public health care facilities as compared to the villages without any public health facilities. Therefore, as a part of reproductive health services, infertility care was highly needed, particularly in the villages where there were no government/public health facilities (Figure 3).

Among the respondents, about 40% mentioned that couples experiencing infertility needed affordable treatment facilities, and about 57% mentioned the facilities of diagnosis and tests at an affordable cost. About three-fourths of respondents stated their need for infertility care and advice within the village, 22% enquired about the presence of a lady doctor at the village health center and about 55% asked to provide proper information related to infertility and treatment. About 43% of respondents mentioned other services needed in rural areas which include counseling of infertile couples, good transport network, male reproductive health specialist, low-priced/discounted medicines, support and care from health workers, financial support to continue the treatment etc. (Table 6).

**Table 6:** Percentage of women mentioned services needed for couples with infertility problem.

Services needed for women with infertility problems*	**Percent**	**Women (*n*)**
Affordable treatment	40.3	64
Facilities of diagnosis and tests at an affordable cost	56.6	90
Infertility care and advice within the village	75.5	120
Presence of a lady doctor at the village health center	22.0	35
Provide proper information related to infertility and treatment	55.3	88
Others	43.4	69

*Multiple answers received.

## Discussions

Infertility is particularly distressing for women who are childless and have strong fertility desires (23). Due to a physical inability to get pregnant, and financial constraints to seek medical help (24), infertile couples get frustrated soon (25). Yet most of the women who experience infertility do not remain childless (23). Timely diagnosis and appropriate treatment can play important role in infertility management (7).

In the present study, it is observed that a considerable percentage of the respondents took a long duration of time in realizing the necessity of fertility treatment and spent a long time seeking treatment. If the causes of infertility problems are diagnosed early, treatment becomes easier. Again, few respondents have not consulted any kind of treatment due to various reasons (26).

Further, the present study shows that women who are more educated and are exposed to media, tend to consult allopathic treatment. The percentage of women who received allopathic treatment is also found higher among those who belong to rich economic status, among Hindus and general and open castes. Similarly, time and money spent on care vary significantly and independently by type of treatment and socioeconomic factors. The limited engagement of the public sector with fertility care and management has been repeatedly noted. Management of infertility is not a priority for the public health sector (27). The present research shows a contrasting picture of fertility care in the study villages. It is observed that a higher percentage of women consulted either AYUSH or no treatment for their fertility issues in the villages without any public health facility which indicates that there is an urgent need for public health intervention targeting women's reproductive health in rural areas.

While India has become popular as a destination for medical tourism and inexpensive assisted reproductive techniques for foreigners, its people, particularly in rural areas, experience a different situation (13, 28). The problem of infertility in India needs to be interpreted in a context of poverty, class and gender inequality and unequal access to healthcare resources (29).

Less proportion of women ended with successful treatment and satisfaction with the process (30). The success rate of getting pregnant among those who consulted allopathic treatment is found highest (above 50%) in the first order treatment and gradually decreases in the successive orders. Better understanding of infertility and how ART works will improve its acceptance in rural areas (31).

A higher proportion of the respondents want their treatment free of cost (32). Further, wide disparities exist in the availability, quality, and delivery of infertility services between the developed and developing nations, as well as between the two regions of the same country (33). Here, it is found that in rural West Bengal, couples cannot opt for expensive allopathic treatment and consult inexpensive alternative treatments. Hence, in the rural health centers, a separate department, equipped with advanced diagnostic facilities and trained and specialized male and female health professionals, is needed to treat sexual and reproductive health problems which will provide inexpensive and quality fertility treatment to the couples (34). Less waiting time in the CHCs, personalized services and provision of medical counseling can also reduce couples' suffering in the quest for conception.

It has been demonstrated repeatedly that traditional healers attract people to come to them for infertility treatment (35–37). Care is often sought from both the formal sector and traditional, system of medicine as is also the case in our populations (38). Despite their affiliation with modern treatment, couples seek varied traditional methods and religious practices in rural areas (39, 40). Further, despite the high prevalence, in rural areas, there is no fertility awareness education which further passes down the common myth, misperception, and negative attitude toward infertility treatment (31).

Despite an attempt to provide data representing India, the present observational research has its limitations in terms of its representativeness. The sampling method used to recruit respondents only includes rural underprivileged population. Although participants were recruited from both villages with and without government health facilities, heterogeneity is still lacking. Subjects represent 20–49 years of women. This may cause the researchers to overlook the subset of subjects who were older than 50 years.

## Conclusions

The cost of infertility treatment largely varies from urban to rural areas, depending upon the type and the phase of treatment consulted. A considerable proportion of women have discontinued their fertility treatment due to various reasons among which unaffordability and inaccessibility of the infertility treatment facility are the foremost. In India, until today no medical insurance covers the medical expenditure on infertility treatment incurred by couples. Hence, the arguments for mandatory insurance coverage may let the state government enact regulations that require varying forms of insurance coverage for infertility treatment. In addition to public services, the private sector and the traditional healer are both important alternative sources of first help. These findings indicate the need to involve healthcare staff from both the formal and informal sectors when investing in the education of infertile couples and the community about infertility.

## Contribution

The present research used primary data which were collected using purposive sampling from the two high infertility prevalence rural districts of West Bengal, India which helped real-time population estimates of infertility. The result was also validated with the prevalence of district-level cross-sectional data (22).

The present research also focused on the factors (mainly high cost of infertility treatment, unavailability of treatment facility, no/low awareness of problem experienced and coping strategies etc.) determining the treatment-seeking behavior of the women ever experienced infertility backed by scientific techniques. A theoretical framework has also been developed to provide a comprehensive overview of the present study which may help future research in Infertility based on unit-level data.

It was the first-ever population-based infertility research in the study area which attempted to fill the research gap by highlighting the current scenario of infertility management and the need for urgent reproductive health services (SDG-3) in the selected districts.

## Data availability statement

The original contributions presented in the study are included in the article/supplementary material, further inquiries can be directed to the corresponding author.

## Ethics statement

The studies involving human participants were reviewed and approved by Students Research Ethics Committee (SREC) of the International Institute for Population Sciences (IIPS). Written informed consent for participation was not required for this study in accordance with the national legislation and the institutional requirements.

## Author contributions

SP contributed to questionnaire preparation, mapping and listing, data collection, data analysis, manuscript preparation, and revision. SU conceptualized the study and guided in the research, manuscript preparation, and revision. All authors contributed to the article and approved the submitted version.

## Conflict of interest

The authors declare that the research was conducted in the absence of any commercial or financial relationships that could be construed as a potential conflict of interest.

## Publisher's note

All claims expressed in this article are solely those of the authors and do not necessarily represent those of their affiliated organizations, or those of the publisher, the editors and the reviewers. Any product that may be evaluated in this article, or claim that may be made by its manufacturer, is not guaranteed or endorsed by the publisher.

## References

[1] Virtala A Vilska S Huttunen T Kunttu K. Childbearing, the desire to have children, and awareness about the impact of age on female fertility among Finnish university students. Eur J Contracept Reprod Health Care. (2011) 16:108–15. 10.3109/13625187.2011.553295 21281094

[2] ESHERE. Physiopathological determinants of human infertility. Hum Reprod Update. (2002) 8:435–47. 10.1093/humupd/8.5.435 12398224

[3] World Health Organization (WHO). International Classification of Diseases, 11th Revision (ICD-11). Geneva: WHO (2018).

[4] Evens EM. A Global perspective on infertility: an under recognized public health issue. In: Carolina Papers in International Health, No. 18. Chapel Hill, NC: University Center North Carolina (2004).

[5] Okonofua F. New reproductive technologies and infertility treatment in Africa. Afr J Reprod Health. (2003)7:7–8. 10.2307/3583339 12816308

[6] Unisa S. Childlessness in Andhra Pradesh, India: treatment-seeking and consequences. Reprod Health Matters. (1999) 7:54–64. 10.1016/S0968-8080(99)90112-X

[7] The Woman's Health Council (WHC). Infertility Treatments for Women-A Review of the Bio-medical Evidence Full Report. (2009), 1–120. Available online at: http://www.rte.ie/news/2009/0923/infertility.pdf

[8] Bunting L Tsibulsky I Boivin J. Fertility knowledge and beliefs about fertility treatment: findings from the international fertility decision-making study. Hum Reprod. (2013) 28:385–97. 10.1093/humrep/des402 23184181

[9] Dyer SJ Abrahams N Hoffman M van der Spuy ZM. Infertility in South Africa: women‘s reproductive health knowledge and treatment-seeking behaviour for involuntary childlessness. Hum Reprod. (2002) 17:1657–62. 10.1093/humrep/17.6.1657 12042294

[10] Disparities in access to effective treatment for infertility in the United States: an Ethics Committee of the American Society for Reproductive Medicine. Fertil Steril. (2015) 104:1104–10. 10.1016/j.fertnstert.2015.07.1139 26364838

[11] Hamilton B McManus B. Infertility Treatment Markets: The Effects of Competition Policy. Washington University Manuscript (2005). p. 1–43. Available online at: http://apps.olin.wustl.edu/faculty/hamiltonb/wpapers/InfertilityTreatmentMarkets.pdf

[12] Jha SN Baur B Haldar A Dasgupta U. A study on fertility perception: an experience from West Bengal, India. Int J Prev Med. (2014) 5:16–20. 24554987 PMC3915468

[13] Adhikari R. Demographic, socio-economic, and cultural factors affecting fertility differentials in Nepal. BMC Pregnancy Childbirth. (2010) 10:19. 10.1186/1471-2393-10-19 20426863 PMC2885993

[14] Singh BP Shukla U. Inability to conceive and treatment-seeking behaviour in Uttar Pradesh state in India. Can Stud Popul. (2015) 42:1–12. 10.25336/P6XC7T

[15] Van Zandvoort H de Koning K Gerrits T. Viewpoint: Medical infertility care in low income countries: the case for concern in policy and practice. Trop Med Int Health. (2001) 6:563–9. 10.1046/j.1365-3156.2001.00756.x 11469951

[16] Cross-Sudworth R. Infertility issues for South Asian women. Diversity in Health and Social Care. (2006) 3:281–7. Available online at: https://diversityhealthcare.imedpub.com/infertility-issues-for-south-asian-women.pdf

[17] Unisa S. Sequence of fertility treatments among childless couples in Ranga Reddy district, Andhra Pradesh, India. Asia-Pac Popul J. (2001) 16:161–76. 10.18356/1958b1a4-en

[18] Ombelet W Cooke I Dyer S Serour G Devroey P. Infertility and the provision of infertility medical services in developing countries. Hum Reprod Update. (2008) 14:605–21. 10.1093/humupd/dmn042 18820005 PMC2569858

[19] Asmabi MA Jithesh MK. Ayurveda management of infertility associated with poly cystic ovarian syndrome: a case report. J Ayurveda Integr Med. (2022) 13:100513. 10.1016/j.jaim.2021.08.006 34980524 PMC8814398

[20] Akbaribazm M Goodarzi N Rahimi M. Female infertility and herbal medicine: an overview of the new findings. Food Sci Nutr. (2021) (9):5869–82. 10.1002/fsn3.2523 34646552 PMC8498057

[21] Jaradat N Zaid AN. Herbal remedies used for the treatment of infertility in males and females by traditional healers in the rural areas of the West Bank/Palestine. BMC Complement Altern Med. (2019) 19:194. 10.1186/s12906-019-2617-2 31366346 PMC6668085

[22] International Institute for Population Sciences (IIPS) (2010). District. Level Household and Facility Survey (DLHS-3), 2007–08: India.

[23] Schwerdtfeger KL Shreffler KM. Trauma of pregnancy loss and infertility for mothers and involuntarily childless women in the contemporary United States. J Loss Trauma. (2009)14:211–27. 10.1080/15325020802537468 21686042 PMC3113688

[24] Cooper GS. An analysis of the costs of infertility treatment. Am J Public Health. (1986) 76:1018–9. 10.2105/AJPH.76.8.1018 3728759 PMC1646651

[25] Greil AL McQuillan J Lowry M Shreffler KM. Infertility treatment and fertility-specific distress: a longitudinal analysis of a population-based sample of US women. Soc Sci Med. (2011) 73:87–94. 10.1016/j.socscimed.2011.04.023 21645954 PMC3126901

[26] Passet-Wittig J Greil AL. Factors associated with medical help-seeking for infertility in developed countries: a narrative review of recent literature. Soc Sci Med. (2021) 5:113782 10.1016/j.socscimed.2021.113782 33895708

[27] Majumdar A Qureshi A. Thinking about infertility from a mixed-methods perspective: the need to look at toxicity in rural India. Sex Reprod Health Matters. (2021) 29:1–7. 10.1080/26410397.2021.1999565 34842497 PMC8923018

[28] Macaluso M Wright-Schnapp TJ Chandra A Johnson R Satterwhite CL Pulver A. A public health focus on infertility prevention, detection, and management. Fertil Steril. (2010) 93:16.e1–10. 10.1016/j.fertnstert.2008.09.046 18992879

[29] Barden-O‘Fallon J. Associates of self-reported fertility status and infertility treatment-seeking in a rural district of Malawi. Hum Reprod. (2005) 20:2229–36. 10.1093/humrep/dei008 15802313

[30] Manna N Pandit D Bhattacharya R Biswas S. A community based study on infertility and associated socio-demographic factors in West Bengal, India. J Dent Med Sci. (2014) 13:13–7. 10.9790/0853-13221317

[31] Harzif AK Santawi VPA Wijaya S. Discrepancy in perception of infertility and attitude towards treatment options: Indonesian urban and rural area. Reprod Health. (2019) 16:126. 10.1186/s12978-019-0792-8 31426818 PMC6700767

[32] Patra S Unisa S. Addressing reproductive health knowledge, infertility and coping strategies among rural women in India. J Biosoc Sci. (2021) 53:557–565. 10.1017/S0021932020000371 32677598

[33] Robert D Nachtigall MD. International disparities in access to infertility services. Fertil Steril. (2006) 85:871–5. 10.1016/j.fertnstert.2005.08.066 16580367

[34] Renckens CNM. Alternative treatments in reproductive medicine: much ado about nothing. “The fact that millions of people do not master arithmetic does not prove that two times two is anything else than four”: W.F. Hermans. Hum Reprod. (2002) 17:528–33. 10.1093/humrep/17.3.528 11870097

[35] Sundby J Mboge R Sonko S. Infertility in the Gambia: frequency and health care seeking. Soc Sci Med. (1998) 46:891–9. 10.1016/S0277-9536(97)00215-3 9541074

[36] Folkvord S Odegaard OA Sundby J. Male infertility in Zimbabwe. Patient Education and Counselling. (2005) 59:239–43. 10.1016/j.pec.2005.08.003 16242296

[37] Stekelenburg J Jager BE Kolk PR Westen EH van der Kwaak A Wolffers IN. Health care seeking behaviour and utilisation of traditional healers in Kalabo, Zambia. Health Policy. (2005) 71:67–81. 10.1016/j.healthpol.2004.05.008 15563994

[38] Dyer SJ Abrahams N Hoffman M van der Spuy ZM. Men leave me as I cannot have children: women‘s experiences with involuntary childlessness *Hum Reprod*. (2002) 17:1663–8. 10.1093/humrep/17.6.1663 12042295

[39] Udgiri R Patil VV. Comparative study to determine the prevalence and socio-cultural practices of infertility in Rural and Urban field practice area of Tertiary Care Hospital, Vijayapura, Karnataka. Indian J Community Med. (2019) 44:129–33. 31333290 10.4103/ijcm.IJCM_172_18 PMC6625263

[40] Laskar MA Goswami P Basak M. A review on traditionally used medical plants for treatment of infertility in North-East India. Int. J. Mod. Pharm. Res. (2020) 4:57–60.

